# Missionary involvement with the Simele massacre in 1933: the end of American sympathy for the Assyrians

**DOI:** 10.1080/13530194.2023.2233218

**Published:** 2023-08-17

**Authors:** Tijmen C. Baarda

**Affiliations:** Leiden University

**Keywords:** Iraq, United States, Assyrians, Christianity, missionaries

## Abstract

The Simele massacre of 1933 in Iraq, in which over 600 Assyrian Christians lost their lives, is an important part of Assyrian national history and is one of the few well-known events in twentieth-century Iraqi history where Christians are involved. While there was a general outcry in Western Europe and the United States against the Iraqi government following the massacre, the American missionaries present locally of the United Mission in Mesopotamia/Iraq did not support the Assyrians following the massacre despite the generally humanitarian approach of their mission and their support for the Assyrian cause in the early years after the First World War. In this article, I argue that apparent apathy from the side of the missionaries was largely the result of a radical change in thinking about missionary involvement in political debates concerning the future of non-Muslim minorities in the Middle East.

## Introduction

The Simele massacre of 1933 was the mass murder of the Assyrian population of the northern Iraqi town of Simele (*Summayl* in Arabic), in which at least 600 Assyrians lost their lives very soon after Iraq gained its independence from Britain in 1932. The massacre is still of a great importance in the national narrative of the Assyrians.^1^^1^Yasmeen Hanoosh, *The Chaldeans: Politics and Identity in Iraq and the American Diaspora* (London: I.B. Tauris, 2019), 86–7. The events at Simele entered Western political discourse almost immediately. Many contemporary European and American newspapers carried reports about the events, which caused much uproar among the general public. This was especially the case in Britain. While there had been earlier harsh reactions to revolts by other ethnic and religious groups—the Kurds and the Shi’is—many now held their government responsible for this massacre against Christians, seeing it as a result of leaving Iraq to its own devices.^2^^2^Hannelore Müller, ‘Assyrian Christians in Iraq, the League of Nations and Transnational Christian Advocacy (1920s–1940s)’, in *Sayfo 1915: An Anthology of Essays on the Genocide of Assyrians/Arameans During the First World War*, ed. Shabo Talay and Soner Ö. Barthoma (Piscataway: Gorgias Press, 2018), 285–6.

The massacre was also an important issue for the United Mission in Mesopotamia/Iraq, a mission founded by American Protestant groups in 1924. The response of these American missionaries is striking and hard to understand: having given full support to the Assyrian cause until the end of the 1920s, they eventually blamed the fact that the massacre happened for an important part on their own behaviour. Despite the strong focus of the mission on humanitarian endeavours more generally, especially education, there were almost no attempts from the side of the missionaries to help the Assyrians after the events. While providing actual relief, such as medical care or provisions for refugees, would have been very difficult given the anti-Assyrian sentiments in Iraqi society right after the massacre, the general sympathy of the missionaries of the United Mission had suddenly almost diminished after the events.

The Assyrians were unpopular with the Iraqi government and among large sections of the Iraqi population during the interwar years. This unpopularity was at its peak in the years immediately before the massacre. A major reason for this was the Assyrians’ participation in the Iraq Levies, the British ground troops in Iraq who had been recruited among the local population to bolster the forces of the resented British authorities.^3^^3^Wilhelm Baum and Dietmar W. Winkler, *The Church of the East: A Concise History* (London: Routledge Curzon, 2003), 142; Sami Zubaida, ‘Contested Nations: Iraq and the Assyrians’, *Nations and Nationalism* 6, no. 3 (2000): 367. Another explanation of the Assyrians’ bad reputation was their resistance to integration into the state of Iraq.^4^^4^This argument is most uncompromisingly advanced by Khaldun S. Husry, ‘The Assyrian Affair of 1933 (I)’, *International Journal of Middle East Studies* 5, no. 2 (1974): 166. While it is correct that many Assyrians—and certainly an important part of the community’s elite—were opposed to integration, this has to be seen in a context where the future of the Assyrians as Iraqi citizens had only gradually materialized, and (crucially) in a country where integration was understood as equal to complete cultural and linguistic (but not religious) assimilation to the Arab majority.

In this article,^5^^5^I would like to thank both the internal and external reviewers who generously took their time to comment upon this article. I place the endeavours of the American missionaries of the United Mission in Mesopotamia/Iraq concerning the Assyrians in Iraq into the wider context of Christianity in the country, arguing that the lack of empathy from the missionaries’ side can be explained by looking at the wider landscape of Christianity in Iraq, as well as developments in Western humanitarian policies in the region. The Assyrians formed only one group within the totality of Christianity in Iraq, but politically they were by far the most active. The missionaries within Iraq were aware of this, and while they were of course generally supportive of the Christians, their missionary archives sometimes show disappointment at a lack of realism on the side of the Assyrians, while the other Christian groups were generally more inclined to work together with the Iraqi government. After setting out the context, I first show how the American missionaries of the United Mission were tightly connected to the Assyrians of Iraq before the massacre. Then, after giving an overview of the events of the massacre, I explain how the actions of the Iraqi army targeted at this specific Christian group can be explained from an Arab nationalist perspective. Finally, I discuss how the attitude of the missionaries after the events had changed dramatically from full support a decade before to irritation and inaction after the events, and how this can be explained by examining political developments in Iraq and changes in missionary and humanitarian thinking. My most important source here is the archive of the United Mission in Mesopotamia/Iraq, which is located in the building of the Presbyterian Historical Society in Philadelphia.^6^^6^Call number RG 89. I would like to thank the Society’s staff for their generous assistance. The archive mainly contains formal reports and letters from the missionaries to the office of the Presbyterian Board of Foreign Missions in New York.

## The Assyrians and their relation to the missionaries

The Assyrians who were affected during the Simele massacre were a specific group of Christians in Iraq. More than other groups of Iraqi Christians, the Assyrians were often victims of discrimination and violence from the Iraqi population and state in the course of the early twentieth century. This had to do with their arrival at the end of the First World War as refugees from the Hakkari mountains and Urmia plains, north of the current Iraqi border, with help from the British. This refugee status, combined with the tribal formation that they retained in its form from before their arrival in Iraq, made them different from the settled Christians.^7^^7^James F. Coakley, ‘The Church of the East Since 1914’, *Bulletin of the John Rylands Library* 78, no. 3 (1996): 181; Ronald S. Stafford, *The Tragedy of the Assyrians* (London: Allen & Unwin, 1935), 82–92. Their most important leader was the patriarch of the Church of the East, who took responsibility for both religious and worldly affairs. From 1920, this office was assumed by Patriarch Mār Shimʿūn XXI,^8^^8^Or XXIII, according to another way of counting. who was only twelve years old at the time, but who was going to play an important political role in the decades that followed. The causes of the Simele massacre can for a large part be traced back to the Assyrians’ history as non-Arab refugees. This is in constrast to the other Christian groups, who during the first half of the twentieth century often identified as Arab and with a few exceptions not as Assyrian.^9^^9^The fact that the Syriac Orthodox in Iraq were influenced by Assyrian nationalism in the period right after the First World War is described by the staunch Arab nationalist Rafāʾīl Buṭṭī, who had a Syriac Orthodox background himself and narrates how he was influenced by this idea before he embraced Arab nationalism. Rafāʾīl Buṭṭī, *Dhākira ʿIrāqiyya*, part 2, ed. Fāʾiq Buṭṭī (Damascus: Dār al-Madá, 2000), 159. Today, the situation is different and identification as Assyrian occurs across all Syriac Christian denominations in Iraq.

With the exception of the Armenian Christians of Iraq, by the twentieth century almost all Christians in the country belonged to one of the four Syriac churches.^10^^10^These are: the Chaldean Catholic Church, the (Assyrian) Church of the East, the Syriac Catholic Church, and the Syriac Orthodox Church. The most important thing that these churches have in common is their use of the Classical Syriac language, but they have been divided into different churches with their own liturgies, ecclesiastical hierarchies and beliefs.^11^^11^See Lucas Van Rompay, ‘The East (3): Syria and Mesopotamia’, in *The Oxford Handbook of Early Christian Studies*, ed. Susan Ashbrook Harvey and David G. Hunter (Oxford: Oxford University Press, 2008), 365–86. However, Arabic has been an important language as well since at least the ninth century C.E. for most of the Syriac Christians, and by the time of the creation of Iraq as a state many Syriac Christians either spoke Arabic or used it in formal situations, and were therefore able to identify as Arabs. This was important, because Arab nationalism was the official state ideology of the Iraqi state even before independence. While state actors, among whom most notably King Faisal, officially appreciated Christianity and Judaism, being accepted as a Christian or Jewish Iraqi citizen was contingent on being and identifying as an Arab.^12^^12^According to the most important ideologue of Iraqi Arab nationalism at this time, Sāṭiʿ al-Ḥuṣrī, all people in Arab countries who spoke Arabic were Arabs. Affiliation with foreign nations was not acceptable. William L. Cleveland, *The Making of an Arab Nationalist: Ottomanism and Arabism in the Life and Thought of Sati’ Al-Husri* (Princeton: Princeton University Press, 1972), 118.

The Assyrian refugees from the Hakkari and Urmia regions were also Syriac Christians. Most of them belonged to the Church of the East (nowadays called the Assyrian Church of the East, in older literature often the Nestorian Church), some were Chaldean Catholic and some others had converted to Protestantism but had a Syriac Christian background. However, for them, identification as Arabs was out of the question. They referred to themselves as Assyrians and not Arabs and their elite propagated the use of their own language, a dialect of Neo-Aramaic called Swadaya.^13^^13^Tijmen C. Baarda, ‘Arabic and the Syriac Christians in Iraq: three levels of loyalty to the Arabist Project (1920–1950)’, in *Arabic and its alternatives: religious minorities and their languages in the emerging nation states of the Middle East (1920–1950)*, ed. Heleen Murre-van den Berg et al. (Leiden: Brill, 2020), 151–7. Several factors are at play here. In their region of origin they already identified as part of the Assyrian nation and this idea was brought to Iraq by the refugees.^14^^14^See for the development of Assyrian nationalism in this region Adam Becker, *Revival and Awakening: American Evangelical Missionaries in Iran and the Origins of Assyrian Nationalism* (Chicago: University of Chicago Press, 2015). Another, equally important factor, was that they came from a region where Arabic was not used. This is in contrast to the Syriac Christians who were already in Iraq at the time of the establishment of the state: many of them spoke Arabic as their native language, especially the elites in the cities of Mosul and Baghdad. Third, having arrived as refugees, the future of the Assyrians in Iraq was unclear for a very long time: until 1937 there were attempts to resituate the community as a whole outside Iraq.^15^^15^For the various attempts to relocate the Assyrians as a group, see Laura Robson, *States of Separation: Transfer, Partition, and the Making of the Modern Middle East* (Oakland: University of California Press, 2017), 83–100. Therefore, while the road towards becoming members of the nation was open to them in theory—by full assimilation and self-identification as Arabs—in practice this was impossible because of the uncertainty of their future as Iraqi citizens. In other words, the Assyrians were so different from the other Syriac Christian groups in terms of origin, language, and political status that their common history and shared heritage as Syriac Christians could not overcome this, with long-lasting consequences.

Another issue was the fact that they were seen as allies of the British because of the participation of Assyrians in the Iraq Levies, providing the disliked foreign rulers in Iraq with ground troops. It was partly because of their different situation without long-lasting ties to Iraq that the British were able to recruit them for this task.^16^^16^Adeed Dawisha, *Iraq: A Political History* (Princeton: Princeton University Press, 2009), 37. Their participation in the Levies had especially caused enmity among the Kurds, since the British used the Levies from time to time against Kurdish rebellions.^17^^17^Ibid. The situation was further complicated because part of the Assyrians actively resisted integration as citizens in Iraq. Their goal was to go back to their region of origin, or otherwise at least receive autonomy within Iraq. While the Iraqi government had proposed their permanent settlement in the north of the country, including Iraqi citizenship and the same rights as the other non-Muslim groups, this was rejected by many leading figures, including the patriarch, because that would be the end of any hope for autonomy.^18^^18^Zubaida, ‘Contested Nations’, 367. One solution proposed during this period by the patriarch’s party was the Assyrians’ mass emigration to Syria, which was still under French control without imminent prospects of independence. They presented this wish to the League of Nation’s Permanent Mandates Commission in 1931.^19^^19^Donabed, *Reforging a Forgotten Nation*, 95–7. In this document, emigration to Syria was presented as an alternative should ‘arrang[ing] our emigration to one of the countries under the rule of one of the Western Nations’ fail. This wish was rejected by the League, but the idea of migrating to Syria remained as Iraqi independence drew nearer.

The issues mentioned above explain why the Assyrians from Hakkari and Urmia were unpopular with both the state and the Iraqi population, and it is important to keep in mind that these issues did not apply to the other Syriac Christians. That does not mean that they did not face discrimination for being Christian, but being able to identify as Arab made that they at least were officially given a chance to be considered loyal Iraqi citizens. It was especially the elite of the Chaldean Catholic Church that took this opportunity.^20^^20^Baarda, ‘Arabic and the Syriac Christians in Iraq’, 157–62. Stronger than that, the Syriac Orthodox journalist and politician Rafāʾīl Buṭṭī did not only embrace the Arab identity of the state,^21^^21^By ‘the Arab identity of the state’ I mean that Iraq was officially an ‘Arab state’, with Arabic as the official language. What this entailed was most clearly defined by Sāṭiʿ al-Ḥuṣrī; see Cleveland, *The Making of an Arab Nationalist*. but even explicitly distanced himself from the Assyrians, as I will show below. It appears that some of the missionaries followed a similar pattern of thought in their developing relationship with the Assyrians.

The American missionaries connected to the United Mission in Mesopotamia (from 1935 United Mission in Iraq),^22^^22^Charles A. Dana and Joe P. Dunn, ‘A Death in Dohuk: Roger C. Cumberland, Mission and Politics Among the Kurds in Northern Iraq, 1923–1938’, *Journal of Third World Studies* 32, no. 1 (2015): 248. which I focus on in this article, had very close ties to the Assyrians in Iraq. The United Mission was a Protestant mission and was founded in 1924 by three American Protestant churches working together and was coordinated by the Presbyterian Board of Foreign Missions.^23^^23^These churches were the Presbyterian Church of the U.S.A., the Reformed Church in America and the Reformed Church in the United States. ‘Guide to the United Presbyterian Church in the U.S.A. Commission on Ecumenical Mission and Relations. Secretaries’ files: United Mission in Iraq’, Presbyterian Historical Society, accessed 20 October 2014, http://history.pcusa.org/collections/research-tools/guides-archival-collections/rg-89. The mission was based in Mosul and was mainly active in the north of Iraq and Baghdad. It came to an end when its schools were seized by the Iraqi government in 1970.^24^^24^Ibid. In Basra, in the south of the country, there was also a station of the related ‘Arabian Mission’ of the Reformed Church in America.^25^^25^See Catherine S. Woodward, ‘The Discourse and Experience of the Arabian Mission’s Medical Missionaries: Part I 1920–39’, *Middle Eastern Studies* 47, no. 5 (2011): 779–80. While the missionaries of the Arabian Mission, which had already been established in the nineteenth century, were related to those of the United Mission, their activities were located outside the area where most Assyrians lived. For that reason I will not discuss them further in this article. Furthermore, there was the Catholic mission of the French Dominicans, which had been largely responsible for conversions to Catholicism from the mid-eighteenth century onwards.^26^^26^Bernard Goormachtigh, *Histoire de la Mission Dominicaine de Mésopotamie et en Kurdistan depuis ses premières origines jusques à nos jours* (Mosul: 1873), 12. Finally, the Anglican Archbishop of Canterbury was in contact with the Assyrians and occasionally intervened by diplomacy in support of the Assyrians, especially after the Simele massacre.^27^^27^The Archbishop of Canterbury had a mission to the Assyrians, that did however not survive the First World War. James F. Coakley, *The Church of the East and the Church of England: A History of the Archbishop of Canterbury’s Assyrian Mission* (Oxford: Oxford University Press, 1992), 336.

## The united mission in Mesopotamia/Iraq and the Assyrians

The United Mission was closely connected to the Assyrians, despite the fact that they formed a very small minority in Iraq. We know this from the mission’s archive in Philadelphia, which contains a wealth of personal letters from the individual missionaries to the office of the American Board of Commissioners for Foreign Missions in the person of Robert E. Speer (1867–1947), the secretary of the American Presbyterian Mission until 1937. The letters give deep insight into the thoughts and experiences of the individual missionaries. Most missionaries were assigned to a particular group of people and specialized in the language of that group. Most letters I refer to below were from Roger C. Cumberland (1894–1938), a young missionary who mainly worked among the Kurds and spoke Kurdish, but who also became deeply involved with the Assyrians around the time of the Simele massacre,^28^^28^For an entire article about Cumberland, see Dana and Dunn, ‘A Death in Dohuk’. and from Dr. R.W. McDowell, an older missionary who was part of the mission together with his wife, knew Syriac and mainly worked with the Assyrians and supported their patriarch even after others started to criticize him.^29^^29^Less is known about McDowell than about Cumberland. For some more details, see Dana and Dunn, ‘A Death in Dohuk’, 250. In this section, I show how the United Mission connected itself to the fate of the Assyrians in Iraq from the start of the mission in 1924.

First of all, the Assyrians formed all of the mission’s local staff throughout the 1920s. This can probably be best explained by the fact that part of the Assyrians were already Protestant before their arrival in Iraq. This situation continued at least until the second half of the 1930s.^30^^30^In 1936, J.W. Willoughby, who we will see later again, wrote in a letter to the New York office from his temporary residence in Iran that the their Assyrian staff was still in Iraq, at a time when most Assyrians were supposed to emigrate from the country (see below). After that I have found no information about the mission’s local staff. Presbyterian Historical Society, RG89–1–15 (1936), 30–11, letter from Willoughby to Speer, Hamadan, 1936. In 1932, Cumberland recognized that this had proven to be a problem in a letter to the office in New York, because of their politically difficult position in Iraq, as he writes that ‘so far as I have been able to see in these nearly ten years, the total impact of the Assyrians on the Muslims of this country forms one of the greatest obstacles to the progress of the gospel’.^31^^31^Presbyterian Historical Society, RG89–1–11 (1932), 30–11. Letter from Cumberland to Speer, Duhok, 17 October 1932. The problematic aspect of all local staff being Assyrians is also mentioned in the ‘Evangelistic Report’ for Dohuk of 1931. Presbyterian Historical Society, RG89–1–10 (1931), 30–2, ‘United Mission in Mesopotamia. Evangelistic Report—Dohuk Field’, 1931.

Also more in general, the mission’s archive is full of references to the Assyrians, concerning both the political aspects and the missionaries’ religious work among them. The other groups of (Syriac) Christians are seldom mentioned. In the beginning, the Assyrians were described in the most favourable terms. Cumberland gave the following description in 1924 in a letter to New York in which he described the different ethnic and religious groups present in Iraq, in which he did not hide his love for the Assyrians:

The fact that in an Arab country, the 4,000 Assyrian soldiers, under British officers, are the acknowledged protection, speaks well and truthfully for their manly qualities. Compared with the other peoples of the country, they are unquestionably the best. Their services are sought as railroad and postal officials, and in private enterprise, because they are the most capable and dependable.^32^^32^Presbyterian Historical Society, RG89-1-3 (1924), 5 March 1924, ‘Selections from a letter written by Rev. Roger C. Cumberland of Semel, Iraq, dated January 9, 1924’. Such essentialist characterizations of the Middle Eastern ‘races’ were more common at the time—similar descriptions could be heard in the British parliament. Husry, ‘The Assyrian Affair of 1933’, part 1, 163.

He was less happy with their religious lives of the Assyrians, questioning whether they could really be called ‘Christians’; not only because they were not Protestant but also because their faith was considered weak and supposedly too much focused on appearances, which is a recurring theme among American Protestant missionaries more in general.^33^^33^Keith David Watenpaugh, *Bread From Stones: The Middle East and the Making of Modern Humanitarianism* (Oakland: University of California Press, 2016), 17. An example can be found in a letter from Cumberland describing an anecdote in which a number of Assyrians told the missionaries they were to convert to Islam if they did not improve their situation, which Cumberland explained as an indication of how weak their faith was. However, he regarded the Assyrians more favourably than the other Christians in Iraq, whose ‘passive attitude towards Islam has brought them to a level with it, or lower’, as he put it in the same letter.^34^^34^Presbyterian Historical Society, RG89-1-3 (1924), 5 March 1924, ‘Selections from a letter written by Rev. Roger C. Cumberland of Semel, Iraq, dated January 9, 1924’.

R.W. McDowell was very much involved with the Assyrians in a political way. He had been in contact with the British authorities since the First World War and who had witnessed the genocide of 1915, and during the course of the 1920s, he actively campaigned for the Assyrian cause. At this point it was by no means decided that the Assyrians were to integrate in Iraq as citizens. A proposal, drawn by McDowell in 1924, shows his views of their future. He was very concerned about the prospect of Britain leaving Iraq, seeing foreign protection of the Christians as an absolute necessity. His plan therefore concerned placing the Assyrians under the direct protection of the League of Nations. In the plan the Assyrians would get their own administrative body, not territorially defined but with jurisdiction over Christians only.^35^^35^Presbyterian Historical Society, RG89–1–3 (1924), 14 February 1924, R.W. McDowell to Robert N. Speer (Presbyterian Board Foreign Missions, NYC). The New York office received the plan favourably, but considered it difficult to implement because of the Arab nationalist tendencies in Iraq.^36^^36^Presbyterian Historical Society, RG89–1–3 (1924), 31 March 1924, Letter from Speer (NYC) to McDowell (Mosul). Indeed, the plan was probably not very realistic, but it shows that the United Mission was at this point actively supporting a lobby for Assyrian autonomy within Iraq.

To understand why the missionaries were so preoccupied with the protection of the Assyrians, we need to consider their deep and essentialist distrust of the Arabs. In 1924 McDowell mentions in a letter to Speer in New York that [t]he Arab is as bitter an enemy of the Assyrians as the Turk and just as dangerous but he is depending upon these Assyrians to save him from the Turk. That once done, the Arab will turn on the Assyrian and rend him.^37^^37^Presbyterian Historical Society, RG89–1–3 (1924), 3 July 1924, McDowell (Mosul) to Speer (NYC). His distrust of the Arabs was shared by Cumberland, who wrote about the ‘city Arabs’ in 1924 that ‘it is impossible to find a good word’.^38^^38^Presbyterian Historical Society, RG89-1-3 (1924), 5 March 1924, ‘Selections from a letter written by Rev. Roger C. Cumberland of Semel, Iraq, dated January 9, 1924’. On the other hand, like Cumberland, McDowell was highly impressed by the Assyrians, which, according to him, are ‘eager to work and when employed are industrious and give better satisfaction than other classes of labour’, as he wrote in a personal report to the New York office a year later.^39^^39^Presbyterian Historical Society, RG–1–4 (1925), McDowell to New York, Personal Report and Departmental Report, 1924–25, received May 25.

The goal of the mission, however, was not to help the Assyrians and other Christians, but to convert Muslims.^40^^40^Presbyterian Historical Society, RG89–1–3 (1924), letter by the Rev. Roger C. Cumberland to Dr. Robert E. Speer, dated 21 June 1924. The official aim was as follows: ‘The aim of the Mission is to evangelize the Mohammedans in the unoccupied area of Mesopotamia, officially designated as Irak. Mosul is a gateway to work among the Moslem Kurds who constitute a new field. Work is to be done among the returning refugees and among the remnant of Jacobite and Chaldean Christians’. Presbyterian Historical Society, RG89–1–4 (1925), ‘Pen Picture of Mosul Station’, Presbyterian Board of Foreign Missions, Department for Specific Work. They were working with the other parts of the population of Iraq, mainly in the north of the country, having established several Arabic- and Kurdish-speaking congregations and successfully running a number of schools. However, in practice, an important and disproportionately large part of their attention was devoted to serving the (Christian) Assyrians. The fact that the mission predominantly worked with people who were already Christian, despite their official goal to convert Muslims and other non-Christians, was shared with other missions in the Middle East. Initial attempts to convert Muslims mostly failed, and missionaries moved their attention to the local Christians.^41^^41^Watenpaugh, *Bread From Stones*, 17. For the Arabian Mission, the number of conversions from Islam is also known to have been very low. Woodward, ‘The Discourse and Experience of the Arabian Mission’s Medical Missionaries’: 783. American Protestant missionaries were often concerned with a perceived lack of faith and making ‘real Christians’ of the believers they encountered.^42^^42^Watenpaugh, *Bread From Stones*, 17. For the missionaries of the United Mission in Mesopotamia/Iraq, there is no reason to assume that they actually managed to convert a large number of people. In practice the conversion of Muslims remained an indirect goal.^43^^43^Presbyterian Historical Society, RG–1–5 (1926), Report on the Assyrian work, 1925–26. Another document describes the lack of success converting the Kurds and Yezidis. Presbyterian Historical Society, RG–1–4 (1925), McDowell to New York, Personal Report and Departmental Report, 1924–25, received May 25. For a large part, this was pursued by the establishment of a number of general educational institutions, which were in the 1920s and early 1930s most notably a number of village schools, a girls’ school in Mosul and a boys’ school in Baghdad, of which the curriculum included general subjects as well as Bible study.^44^^44^Presbyterian Historical Society, RG89–1–4 (1925), Presbyterian Board of Foreign Missions, Department for Specific Work, ‘Pen Picture of Mosul Station’. Most of these schools were open to Muslims, although most students were Christian.^45^^45^Presbyterian Historical Society, RG89–1–5 (1926), Report on Mosul Americal School for Girls, 1926; and Rev. Calvin K. Staudt, Ph.D., ‘Moslems in the Americal School for Boys in Baghdad’, 8 March 1926. Educational endeavours were explicitly set up in order to assist evangelization, but to what extent it was ‘evangelistically profitable to carry education’ was open to debate.^46^^46^Presbyterian Historical Society, RG89–1–6 (1927), ‘Some Reflections on Conditions in the United Mission in Mesopotamia on the Third Anniversary of its Establishment’, dated (uncertainly) to 1927. However, as with other American Protestant missions, missionary educational endeavours can also be interpreted as fully-fledged humanitarian projects.^47^^47^Watenpaugh, *Bread From Stones*, 4; Andrew Preston, ‘Faith and empire: American missionaries, humanitarianism, and the spread of human rights’, in *Civil Religion, Human Rights and International Relations: Connecting People Across Cultures and Traditions* (Cheltenham: Edward Elgar, 2012), 106. In fact, Heather Sharkey argues that from the 1930s onwards education became the primary goal in American missions in the Middle East as evangelization became almost impossible for political reasons.^48^^48^Heather J. Sharkey, *American Evangelicals in Egypt: Missionary Encounters in an Age of Empire* (Princeton: Princeton University Press, 2008), 139–40.

Since the Assyrians who had come from Hakkari and Urmia were partly Protestant, there was an Assyrian Protestant congregation in Mosul, which the United Mission supported from its start in 1924. However, in a letter of the same year to the New York office, McDowell expressed his concern that the Assyrians be weakened if they support the Protestant Assyrians too much:

The unsettled political conditions and our desire to minister to the people of the Old Church without weakening it have been the reasons why we have refrained from organizing this Assyrian congregation into an Evangelical body.^49^^49^Presbyterian Historical Society, RG–1–4 (1925), McDowell to New York, Personal Report and Departmental Report, 1924–25, received May 25.

The reason why McDowell was so cautious is that by creating an ‘evangelical body’ for the Protestant Assyrians, this might weaken the Old Church, which was the name they used for the Church of the East, to which the majority of the Assyrians belonged, and of which the earlier-mentioned Patriarch Mār Shimʿūn XXI was the head. The line of thinking here appears to be that a weaker Church of the East could result in a weaker negotiation position for political aims such as autonomy in Iraq.

The more time passed, the less realistic special provisions for the Assyrians however became: no emigration and no special treatments, certainly no separate administrative body. Though the patriarch and the people around him did not change their demands, the missionaries began to redirect their attention to supporting the settlement of the Assyrians in Iraq, and decided to start a formal Assyrian Evangelical congregation in 1926, according to an anonymous report.^50^^50^Presbyterian Historical Society, RG–1–5 (1926), Report on the Assyrian work, 1925–26. The same document shows that they still supported the (non-Protestant) Church of the East as well, hoping that the future would bring about a ‘renewal of Christian activities’.^51^^51^Ibid. The document shows optimism about the settlement of the Assyrians in Iraq, in contrast to the concerns uttered by McDowell in 1924 about what would happen if Iraq became independent, as cited above.^52^^52^Ibid. However, while the missionaries kept supporting the Church of the East, they began to disagree with the church’s continued policy of demanding autonomy in Iraq. This is most visible in the missionaries’ changing views on the patriarch, who vocally expressed this policy. The first expression of concern from the side of the missionaries that I found comes from 1927, when an anonymous ‘Report on work for Assyrians’ spoke about the fact that part of the Assyrian leaders insisted that ‘they will not settle permanently in Iraq to become politically subject to the Moslem Arab rulers’, to be either protected by the British or taken out of the country. At the same time, however, the report acknowledges that these leaders ‘do not speak altogether without reason’, and also the patriarch, who was still only 19 years old and had recently returned from Britain where he had completed his education, was praised for being ‘intelligent, modest, but with a quiet dignity’.^53^^53^Presbyterian Historical Society, RG–1–6 (1927), Report on work for Assyrians, April 1^st^ to December 31^st^, 1927. There is another document from the same year by James W. Willoughby favourably describing the young patriarch, describing the way he was preaching in Syriac and especially that his sermons focused on ‘the necessity of spirituality in religion, and the futility of fasting ritual and such things in themselves, if not undertaken with earnestness, and a discerning soul’, which addressed one of the missionaries’ main criticisms of the faith of the Church of the East. Presbyterian Historical Society, RG–1–6 (1927), 30–11, ‘The Boy Patriarch. Now Less Boy and More Patriarch’. As the patriarch became older, however, he became more involved in politics and multiple missionaries show that they were not happy with his continued agitation in favour of autonomy. Especially Cumberland became very explicitly annoyed with the behaviour of the patriarch. In 1931, he wrote in this context in a letter to New York that ‘[i]n my eight years of acquaintance with the house of Mar Shimon, its most prominent characteristic has been ambition for personal aggrandizement, with not the slightest care for the people except as they may serve that end’.^54^^54^Presbyterian Historical Society, RG–1–10 (1931), 30–11, letter from Cumberland in Duhok to Chamberlain, 22 April 1931. In 1932, he accused the patriarch again in a letter to Speer and made clear that he saw cooperation by the Assyrians with their neighbours as the way forward:

As for the situation of the Assyrian group as a whole, it is bad, due almost altogether to the bad leadership of Mar Shimon. His political aspirations have won for his people the distrust if not the hatred of both Arabs and Kurds, and the outlook for the future is very dark. So long as they are in Iraq and they maintain their present attitude of opposition to all their neighbors, there will be trouble.^55^^55^Presbyterian Historical Society, RG89–1–11 (1932), 30–11. Letter from Cumberland to Speer, Duhok, 17 October 1932.

McDowell, on the other hand, did not drop his support of the patriarch. In 1932, when asked to give information to Speer in the New York office about the Assyrians and their relationship to the patriarch, he who wrote much favourably about him and that he was ‘spiritually alive, of high moral character, fair mental equipment and concerned with the spiritual welfare of his people’.^56^^56^Presbyterian Historical Society, RG89–1–11 (1932), 30–11. Letter from Mr. McDowell to Speer, 13 January 1932. In this letter, McDowell did not write about the position of the patriarch in relation to the political settlement at all, although he repeated his view that the Assyrians and other minorities would be in need of international protection after the departure of the British.^57^^57^Ibid.

It is not clear from the mission’s archive if the mission had an official opinion about the patriarch at this point. But what is clear is that the mission still actively supported the Assyrians in general. In addition, the members of the mission’s local staff were still all Assyrian, but these staff did not support the political policies of the patriarch.^58^^58^Presbyterian Historical Society, RG89–1–12 (1933), Evangelistic Report—Dohuk Field – 1933. Sargon Donabed notices that the Assyrians who did not follow the patriarch’s policies were indeed influenced by Protestant Assyrians. Donabed, *Reforging a Forgotten History*, 102. The special support to the Assyrians however came to an end when the United Mission decided to refocus on the general population of Iraq, which happened gradually after the Simele massacre of 1933.

## The Simele massacre of 1933

The fact that the Simele massacre took place shortly after Iraq gained its independence in 1932 is not a coincidence. In 1930, the year of independence was fixed by a new treaty between Britain and Iraq.^59^^59^Peter Sluglett, *Britain in Iraq: Contriving King and Country* (London: I.B. Tauris, 2007), 123–24. Many Assyrians were afraid that independence would mean the end of their protection by the British, making their life in Iraq impossible because of their reputation and the role of the Assyrian Levies as an army for the British.

A key narrative of the Simele massacre was written down by Lieutenant-Colonel R.S. Stafford, the British administrative inspector for Mosul, who witnessed the events at close hand. His book is a detailed account of what he saw, did, and thought, but it is not an academic work. Major research has been provided by Khaldun Husry, in defence of the Iraqi government, Sami Zubaida, mainly based on British Foreign Office documents, and more recently by Sargon Donabed, who added more Assyrian sources, including oral testimony.

When the Assyrian Levies were disbanded in 1933 because of their relationship to the British, the former soldiers were allowed to retain and carry weapons in order to be able to defend themselves against the Kurds among whom they lived.^60^^60^Husry, ‘The Assyrian Affair of 1933 (I)’, 172. In early 1933, an Assyrian tribal leader and formal soldier in the Levies called Mālik Yāqū started a campaign to oppose the integration of Assyrians into Iraq as citizens with the same rights as other minorities.^61^^61^Solomon (Sawa) Solomon, *The Assyrian Levies* (1996), 33. He did so in cooperation with the patriarch and by touring with armed men around the villages in the north.^62^^62^Stafford, *The Tragedy of the Assyrians*, 110–11; Husry, ‘The Assyrian Affair of 1933 (I)’: 170; Donabed, *Reforging a Forgotten History*, 100. This led to a hostile response of the Iraqi government and a deterioration of public opinion with respect to the Assyrians.^63^^63^Stafford, *The Tragedy of the Assyrians*, 129. The Iraqi army then dispatched troops to the area.^64^^64^Husry, ‘The Assyrian Affair of 1933 (I)’: 172; Donabed, *Reforging a Forgotten History*, 101. When a negotiation between Yāqū and the army did not succeed, Yāqū with a group of armed men decided to leave the country and cross the border into French-controlled Syria, in line with the above-mentioned proposal by the patriarch’s party to emigrate there. They were reportedly joined by 800 other Assyrians from various tribes. Syria sent them back and Iraq accepted their return on the condition of first being disarmed by the authorities in Syria; however, this disarmament did not occur.^65^^65^It is unclear why the French had not disarmed the Assyrians or had even returned their weapons. Husry, ‘The Assyrian Affair of 1933 (I)’: 174. Donabed adds that the French did so because the weapons had been given to the Assyrians by the Iraqi state in the first place. Donabed, *Reforging a Forgotten History*, 106. When the Assyrians crossed the border back to Iraq in the beginning of August, fierce fighting broke out between the Assyrians and the Iraqi army,^66^^66^It is uncertain who started the fighting—the Iraqi army might have opened fire when they saw that the Assyrians were unexpectedly armed, but official British reports suggest that the Assyrians had started the fighting. For the argument that the fighting was started by Yāqū and his party, see Husry, ‘The Assyrian Affair of 1933 (I)’: 175. Donabed holds that the army started fighting as soon as they saw the Assyrians return. Donabed, *Reforging a Forgotten History*, 106. which lasted almost a full day, in which the Iraqi army eventually got the upper hand.^67^^67^Stafford, *The Tragedy of the Assyrians*, 131–2; Donabed, *Reforging a Forgotten History*, 106. Afterwards, most Assyrians fled back to Syria, while others tried to go back to their villages. The army then started a campaign to capture the fleeing Assyrians because they were still armed. Many villagers fled to the Assyrian town of Simele on 8 August. After three days, the army massacred all men in Simele, while they tried to prevent the women and children from being killed.^68^^68^Stafford, *The Tragedy of the Assyrians*, 144–6; Donabed, *Reforging a Forgotten History*, 109–16.

After the events in July and August 1933, the Iraqi army received a warm welcome in Baghdad at the end of August upon their return from the north.^69^^69^Stafford writes, however, that the crowds welcoming the army in Baghdad were organized by the government ‘by the spending of a few pounds’, but that similar demonstrations took place in Mosul that were genuinely popular. Stafford, *The Tragedy of the Assyrians*, 162 and 164. It should be noted that at this point the massacre in Simele was not acknowledged, and that the people were cheering for the defeat of the Assyrians who had fought the army.^70^^70^Zubaida, ‘Contested Nations’, 371. In August, in various places in Iraq, especially Mosul, anti-Assyrian attacks, as well as attacks against the British and French residents, took place.^71^^71^Stafford, *The Tragedy of the Assyrians*, 165. Immediate relief, including some medical help and transfer to new temporary camps, had come only days after the massacre and was provided for by the Iraqi government.^72^^72^Ibid., 147.

In the period between 1933 and 1937, a collective movement of the Assyrians out of Iraq was back on the table, which was reportedly supported by around 90% of the Assyrians at the time.^73^^73^Ibid., 176. With support by the League of Nations, preparations for resettlement in British Guiana were made, but this could not be realized. The option for settlement in Syria failed, too, although a few thousand Assyrians were allowed to settle there and received Syrian citizenship.^74^^74^Robson, *States of Separation*, 99. In 1937 this idea was eventually abandoned when the Iraqi government declared to the League of Nations that the Assyrians who remained in the country would remain there as citizens but without any special rights.^75^^75^Müller, ‘Assyrian Christians in Iraq’, 298. The Assyrians had no choice other than accepting that, because the patriarch and main proponent of resisting integration had left the country and the former Levies were not armed anymore.

The position of the Assyrians in Iraq had changed. It was the end of their years in a tribal, partly armed formation with the hope for autonomy. The government forced the patriarch to leave the country out of fear of armed resistance and moved him with his family to Cyprus, from where, in 1940, he moved to Chicago. The official policy of the Assyrian Church of the East to aim for far-reaching autonomy or independence finally changed in 1948, when Patriarch Mar Eshai Shimʿūn wrote that the Assyrians in the Middle East should be loyal citizens in the various countries. This was an about-turn in policy in comparison to the patriarch’s earlier insistence on autonomy.^76^^76^Heleen Murre-van den Berg, ‘Light from the East (1948–1954) and the Deterritorialization of the Assyrian Church of the East’, in *Religion beyond its Private Role in Modern Society*, ed. Wim Hofstee and Arie van der Kooij (Leiden: Brill, 2013), 121–8.

## The massacre: purely political or anti-Christian?

Although the massacre was initially officially denied by the Iraqi government,^77^^77^Khaldun S. Husry, ‘The Assyrian Affair of 1933 (II)’, *International Journal of Middle East Studies* 5, no. 3 (1974): 345. there is no doubt among historians about the atrocities that happened in Simele, but the extent to which the Iraqi government is to blame is still debated. These controversies concern two issues.^78^^78^For the development of the academic discourse surrounding the Simele massacre, see Heleen Murre-van den Berg, ‘Writing Assyrian History: The Military, the Patriarch and the British in Yaqu bar Malek Ismael’s *Assyrians in Two World Wars* (Tehran 1964)’, in *Sayfo 1915: An Anthology of Essays on the Genocide of Assyrians/Arameans during the First World War*, ed. Shabo Talay and Soner Barthoma (Piscataway: Gorgias Press, 2018), 221–4. First, while a response by the Iraqi army to the armed campaign by the party of Yāqū was inevitable, a major question among historians is whether the Iraqi government intended to commit a massacre on Assyrian civilians, or that the army only intended to punish Yāqū and his party for his revolt but that a massacre was a more or less spontaneous act of individuals within the army.^79^^79^Stafford claims that it was planned by Bakr Ṣidqī, while Husry (in response to Stafford) writes that it is more probable that it was an irregular action by the army division in question. The second issue is whether the Assyrian leadership could justify its refusal to accept the government’s settlement and integration proposal. Husry is extremely severe on this point, maintaining that the Assyrians had a comfortable life in Iraq and no cause for complaint.^80^^80^Husry, ‘The Assyrian Affair of 1933 (I)’, 164. Stafford, in his eyewitness-testimony, shows more sympathy for the Assyrians, but concentrates on the unrealistic expectations of the patriarch and the sincere efforts of the Iraqi government. Donabed rightly stresses anti-Assyrian sentiments in Iraq, as well as unrealistic housing plans, justifying the anti-integration campaign and the emigration attempt.^81^^81^Donabed, *Reforging a Forgotten History*, 99–100. An important part of Stafford’s argument is that, while it should have never ended in a massacre, the way in which the government treated the Assyrians in general was fair to say the least.^82^^82^Stafford, *The Tragedy of the Assyrians*, 128. This is in line with the fact that in general, the British supported the Iraqi government in their treatment of the Assyrians and their refusal to grant the Assyrians special rights or autonomy,^83^^83^Zubaida, ‘Contested Nations’, 377. despite the suspicions in Iraq that the British were aiding the Assyrians against Iraq in order to delay independence.^84^^84^Stafford, *The Tragedy of the Assyrians*, 93; Husry, ‘The Assyrian Affair of 1933 (II)’, 346 and 350. Laura Robson criticizes the British government and argues that the Assyrians were used in order to secure the continued British control over Mosul, among other things, and then let down.^85^^85^Robson, *States of Separation*, 52.

Both the Iraqi and British governments saw the massacre as a result of a political issue and denied that it had anything to do with the fact that the Assyrians were Christian. That is also the position of Stafford. This makes sense in the light of their special position in Iraqi society that I discussed above, although it seems probable that anti-Christian sentiments also played a role. Many Europeans, including the Archbishop of Canterbury, saw the events as an anti-Christian attack.^86^^86^Husry, ‘The Assyrian Affair of 1933 (II)’, 353. There are indeed some indications suggesting that the anti-Assyrian sentiments during the aftermath of the Simele massacre were also directed at the Christian population in general, although there is not enough evidence to proof this.^87^^87^Sargon Donabed mentions that people were threatened with death if they did not convert to Islam immediately. Donabed, *Reforging a Forgotten History*, 110–11. Stafford also mentions the general anti-Christian sentiments, without however providing details. Stafford, *The Tragedy of the Assyrians*, 167. If the sentiments were indeed anti-Christian in general, this might explain why the Chaldean Catholic Church, in the issue of its official journal *al-Najm* appearing a month after the events, did not mention the massacre but opted to remain silent and instead praise the newly crowned King Ghazi, even though he is known to have had a major role in the massacre while he was still Crown Prince.^88^^88^*Al-Najm* 5:7 (1933). For Ghazi’s role in the Simele massacre, see Donabed, *Reforging a Forgotten History*, 117. According to this explanation, the Chaldeans distanced themselves from the Assyrians and expressed their loyalty to the King and the Iraqi state even more forcefully to prevent trouble for themselves. On the other hand, it is not unthinkable that some Syriac Christians genuinely accepted the government’s interpretation. This was the position of Rafāʾīl Buṭṭī, a staunch Arab nationalist with a Syriac Orthodox background, who used the words ‘the Assyrian campaign’ (*al-ḥamla al-Ashūrī*) when he spoke about the events in his memoirs, without even mentioning the overreaction of the army and blaming the Assyrians for everything that happened.^89^^89^Rafāʾīl Buṭṭī, *Dhākira ʿIrāqiyya*, part 2, 159. He writes here, in defence of accusations that he was an Assyrian himself: ‘[T]he government of Iraq had granted a vast land [to the Assyrians], and the well-known events of 1933 happened (the campaign of the Assyrians)’.

## A change in attitude of the United mission missionaries

The mission’s archive in Philadelphia does not feature any material written during the summer in which the massacre happened. However, from a body personal letters by Roger Cumberland—not part of the archive but published by Charles A. Dana and Joe P. Dunn in 2015 – we know that he personally spoke out against the Iraqi army and that he ‘allowed widows and orphans of the massacre to take refuge in his yard in Duhok’.^90^^90^Dana and Dunn, ‘A Death in Dohuk’, 257–8. Because of this, the government targeted the United Mission as ‘anti-Iraqi’ and foreign, and sent Cumberland even out of Duhok for allegations of political activity under the guise of his work as a missionary.^91^^91^Annie Tracy Samuel, ‘The Open Door and U.S. Policy in Iraq between the World Wars’, *Diplomatic History* 38, no. 5 (2014): 938–9; Stafford, *The Tragedy of the Assyrians*, 140. Despite Cumberland’s actions, which were courageous and had put himself and the mission in danger, the missionaries clearly held the Assyrian leadership to a large extent responsible for what happened. Remarkably, in an anonymous ‘Evangelistic Report’ of 1933 to the office in New York, the mission expressed the support of the government’s position here and did not explicitly blame it for the atrocities:

On the other hand, we see the patience of Government eventually exhausted (I think no other government in the world would have been patient so long) and the forces of fanaticism in control, culminating in the massacre at Semeil. We see the patriarchal program promoted without regard for truth: baseless rumors were spread as to the prospects of Assyrian emigration or the establishment of their autonomy in this country, and the basest slanders of the Government were circulated, all for the purpose of preventing settlement and so to appeal to the League and the ‘Christian’ nations of the West to rescue them.^92^^92^Presbyterian Historical Society, RG89–1–12 (1933), Evangelistic Report—Dohuk Field – 1933.

In other words, according to this rapport the massacre was not the government’s fault, but caused by a combination of fanaticism from the side of the Iraqi population and a failing and dangerous strategy of the Assyrian leadership to resist integration in order to be ‘rescued’ by western countries.

The actions of Cumberland following the massacre caused allegations against the mission from the side of the government, but according to the mission this allegation could not be justified because even before the massacre Cumberland had been highly critical of the Assyrian leadership, which, as we saw, is correct.^93^^93^This is also the interpretation of Stafford, who wrote that ‘[s]uch an accusation was absurd’, because a few months before he ‘had greatly annoyed the Assyrians by an outspoken article in an American magazine, in which he had pointed out that all the alleged grievances of the Assyrians were in fact not well grounded’. I was not able to trace this article. A good relationship was however quickly restored. In 1933 John S. Bodeau wrote in a ‘mission narrative’ to the office in New York: ‘the Government recognizes our integrity as religious workers, and our genuine concern for the national aspiration of the country’, and went on to lament the death of King Faisal, ‘a ruler who stood above religious, political and racial parties, whose influence was ever for tolerance and progress’.^94^^94^Presbyterian Historical Society, RG89–1–12 (1933), 30–2. United Mission in Mesopotamia—Mission Narrative for 1933, by John S. Bodeau, whom I have not been able to identify. This statement shows not only that the mission was again accepted by the Iraqi government, but also that the mission started to fully support the government and its policies towards the minorities of the country. These warm words about an Arab government are in sharp contrast to the harsh words about Arabs that were uttered less than ten years earlier by missionaries in the beginning phase of the mission, as I pointed out above.

At the same time, it appears that the United Mission even started to distance itself from efforts by many Assyrian leaders to preserve their Assyrian identity as a separate ethnic group in Iraq. Shortly before the Simele massacre, the missionaries already started to drop their support of the aspirations of Assyrian autonomy or independence, but after the massacre the missionaries started to see their identification as a national minority as undesirable in their new situation as Iraqi citizens. This becomes clear in the context of the reduction of subsidy to the Evangelical Assyrian school in Baghdad. This school had been established by McDowell and his wife in 1921, before the start of the United Mission, in order to help them as refugees.^95^^95^Presbyterian Historical Society, RG89–1–10, ‘[s]hort report about the spiritual and educational work carried on among the Assyrian refugees in Baghdad by the Assyrian Presbyterian Evangelical Church’, attached to a letter from Rev. Khendo H. Yonan (pastor for the United Mission) to Speer, New York City, 17 April 1931. At that time, the residence of the Assyrians in Iraq was still widely regarded as temporary, so the establishment of a school especially for the Assyrians was not controversial. The school became an official part of the United Mission, and the mission decided to reduce their subsidy in 1930, this was in an effort to make it self-sustainable and not because it was not considered a worthwhile institution.^96^^96^The 1931 mission report states: ‘So far as our budget is concerned the change goes into effect with the beginning of 1932, but if the Assyrian Community can sustain its school independently of our help, we wish them all success’. Presbyterian Historical Society, RG89–1–10, ‘U.M.M. 1931 Report’, 5. In 1936, the mission decided to cancel the subsidy entirely. The reasons for that are given in a report about statements by James W. Willoughby, a missionary who had been part of the mission since the start in 1924:

[The mission] did not consider that it should subsidize a foreign language group in Iraq. The Assyrians have never adapted themselves to the life in Iraq but have continued their school and their church in their original language. The Government is desirous of having them absorbed into the Iraqi nation. The Mission feels that its primary purpose is to evangelize the Arabs. Consequently it felt that it could not continue paying out money to a non-Arab community.^97^^97^Presbyterian Historical Society, RG89–1–16, ‘Memorandum on conversation with Mr. Willoughby regarding the Assyrians in Baghdad and his comments on a letter from Mr. Khendo H. Yonan with regard to the situation there’ (1937). While this document is from 1937 it explains the decision to cancel the subsidy that was taken in 1936.

The subsidy was now cancelled because it served a ‘foreign language group in Iraq’, which the mission was not meant for. Formally, it is indeed correct that the mission’s goal had always been the conversion of Muslims, but in practice this is a sharp break with the past. In addition, the statement also includes a reproach that the Assyrians did not integrate into Iraq. Khendo H. Yonan, the Assyrian pastor who was responsible for the school, complained against this decision, and defended the loyalty of the Protestant Assyrians to Iraq in a letter in 1936: ‘Of course we are not an Arabic speaking community, but we are by far more Iraqis in spirit and in every way than the other sects among the Assyrians here in Baghdad’.^98^^98^Presbyterian Historical Society, RG89–1–15, letter from Khendo H. Yonan to Dr. Coan, 6 August 1936. By ‘other sects among the Assyrians’, he means non-Protestant Assyrians, especially the Assyrians of the Church of the East, which was the majority. However, in a letter from B.D. Hakken, the secretary of the mission since 1937, to Khendo Yonan, this line of argumentation was rejected:

The Mission has never felt that it was responsible for the school and especially at this time it seems to be a needless expense since there are good Government schools and your children would not lose out educationally if you dropped the idea of conducting a school. You possibly feel that you must teach the Syriac tongue to your children, but is this the wisest course? The language of the country is Arabic and we feel that you should put emphasis on your children learning this language. This would be one way of identifying yourselves with the people of this country if you intend to remain citizens of Iraq.^99^^99^Presbyterian Historical Society, RG89–1–17, letter from B.D. Hakken (secretary of the mission) to Khendo H. Yonan, 1 November 1938.

In other words, not only did the mission want to withdraw their responsibility from the school, the secretary here even says that their school could better be closed down altogether. The letter shows a very sharp rejection of teaching the Neo-Aramaic language and an urgent call on the Assyrians to assimilate to the Arab majority culture: loyal citizenship was not enough. More than that, the Arab government of independent Iraq was now portrayed positively and the quality of state education praised.

## Missionary apathy in relation to interwar humanitarianism

With the exception from the intervention by Cumberland that I mentioned above, there was no serious effort from the side of the United Mission to help the Assyrians after the massacre. On the contrary, despite their substantial involvement among the Iraqi Assyrians, in the years after the massacre they started to focus on other, non-Christian communities whom the missionaries deemed to be more in need of their attention. How could it be that this community could not count on more compassion from the missionaries who had supported them from the beginning?

The change coincides with rapid political changes in Iraq. In the early 1920s, Britain was still in charge in Iraq and maintained a close connection to the Assyrians. At that point in time, the Assyrians were still refugees and their future was undecided. From the 1930s the country was on its way to independence and now the government expected from the Assyrians that they integrate into Iraq as citizens. This change of government and change of policy concerning the Assyrians was apparently followed by the missionaries of the United Mission, who were forced to deal with a new political reality. A parallel can be made here with the history of Near East Relief (NER), which was also founded by Presbyterians and served Armenian refugees from the Anatolian genocide in Syria and Lebanon—an example that Keith Watenpaugh uses to trace the development of modern humanitarianism in the Middle East. Watenpaugh writes that after the genocide, the efforts of this organization first focussed on the Armenians’ relocation to southern Anatolia, and when this region fell out of French hands and had to be evacuated, their permanent establishment in Syria and Lebanon. In the first period, much of their work consisted of the establishment of schools, where the students did not learn Arabic, because their policy was to preserve the community’s Armenian identity.^100^^100^Watenpaugh, *Bread From Stones*, 113–5. The NER genuinely believed in this policy, but it went hand in hand with the French mandatory policy of keeping the Armenian population separate from the Arab population as a force against rising Arab nationalism. In the second period, at the end of the 1920s, the focus clearly shifted to integration of the Armenians in Arab society, without going against the policies of the local governments.^101^^101^Ibid., 191. Thus, like NER, the United Mission followed the policies of the new government of Iraq as it took power of the country, perhaps because they were dependent on the government for their operations.

Following the policies of the Iraqi government must have been quite a step, considering the missionaries’ initial highly negative attitude towards the country’s local population and especially the Muslim Arabs as shown above. However, on a more global scale we see American Protestant missionaries change their attitudes towards Islam from the late 1920s and especially in the 1930s.^102^^102^Thomas S. Kidd, *American Christians and Islam: Evangelical Culture and Muslims from the Colonial Period to the Age of Terrorism* (Princeton: Princeton University Press, 2009), 75–95. This was partly the outcome of a rising influence of modernists (as opposed to fundamentalists) in American Protestantism, which had a serious impact on the practice of various missions in the Middle East. This is also the case for the American Protestant mission in Egypt, which evidently showed a growing appreciation of Islam from the 1920s, and a new emphasis on social service, such as education, without an explicit goal of conversion.^103^^103^Heather J. Sharkey, *American Evangelicals in Egypt: Missionary Encounters in an Age of Empire* (Princeton: Princeton University Press, 2008), 103 and 139–41. This appreciation came despite a concurrent wave of Egyptian anti-missionary sentiments in the period 1928–1933.^104^^104^Samir Boulos, *European Evangelicals in Egypt (1900–1956): Cultural Entanglements and Missionary Spaces* (Leiden: Brill, 2016), 57. As for the United Mission in Mesopotamia/Iraq, the archival materials do not explicitly indicate Islam being held in higher esteem or a rethinking of evangelization, but since the missionaries in Iraq came from the same Presbyterian circles as in Egypt, it explains their trust in the Iraqi Arab (and largely Muslim) authorities from the 1930s. Even Roger Cumberland, who still spoke so harshly about Muslims and Arabs in the 1920s (see above), lamented in a letter to Speer in New York in 1932 the ‘distrust if not the hatred of both Arabs and Kurds’ on the part of many Assyrians, which shows that he himself had fundamentally changed his views on the Arabs by the 1930s.^105^^105^Presbyterian Historical Society, RG89-1-11 (1932), letter from Cumberland to Speer, Duhok, 17 October 1932.

A final factor worth exploring is the involvement in politics and diplomacy of the missionaries, who often had a ‘conspicuously political role’ in providing the State Department with information, as Annie Tracy Samuel writes in her article about American policy in Iraq in the interwar years.^106^^106^Samuel, ‘The Open Door and U.S. Policy in Iraq between the World Wars’, 938. The United States had not joined the League of Nations and had no immediate authority in Iraq, but expected the British-led mandate and later the independent Iraqi state to offer Americans the same opportunities in terms of business, religion and education as it did Europeans.^107^^107^Ibid., 931. When the missionaries tried to support emigration programmes for the Assyrians in the 1920s, they therefore communicated with the League of Nations rather than with the American government. After the Simele massacre, however, American missionaries of the United Mission—including Cumberland—met with hostility from the side of the Iraqi government, and therefore American interests were compromised. Samuel notes in this respect that the American government did not reproach the Iraqis for the massacre in order to retain a good relationship and prevent hostility to Americans from escalating.^108^^108^Ibid., 938–9. It could be argued that the similar response to the massacre by the missionaries was motivated by the same considerations of American foreign policy: a good relationship with the mission was essential for the continuation of their programme. That can only be part of the explanation, though: the internal documents cited above show that the lack of support was in line with a genuine change of thinking about the Assyrian leadership.

Notwithstanding the explanations above, it remains a fact that the United Mission in Mesopotamia/Iraq dropped their support for the cause of the ethnic and religious minority of Assyrians in less than a decade due to external factors. Put in bluntly cynical terms, the missionaries had lost interest in the Assyrians and there was no longer any reason to support this group. This idea is supported by Keith Watenpaugh’s conclusion in relation to the NER’s changing attitudes to the Armenians in Syria: [T]he interwar understanding … of why certain categories of people should or should not receive humanitarian assistance often had very little if anything to do with the protection of their human rights per se and instead usually had more to do with their ethnicity, religion, citizenship, and utility to states and ideologies.^109^^109^Keith David Watenpaugh, ‘Between Communal Survival and National Aspiration: Armenian Genocide Refugees, the League of Nations, and the Practices of Interwar Humanitarianism’, *Humanity: An International Journal of Human Rights, Humanitarianism, and Development* 5, no. 2 (2014): 176.

## Conclusion

Given the general support for the Assyrians in Europe and at the League of Nations, and the public outcry as the world got to know what happened in Simele, the American missionaries of the United Mission would intuitively be expected to have acted in their defence. This is especially hard to understand because of their tight ties to the Assyrians and the fact that their local staff consisted of Assyrians. And even though the political situation around the time of the Simele massacre may have forced them to drop support of the Assyrian patriarch, in the years after the massacre they went much further by no longer supporting the Assyrians and their efforts to preserve their own identity and language. While this change in policy was a gradual process, it is likely that it was accelerated because of the Simele massacre.

The ecclesial make-up of Iraqi Christianity shows that the country hosted other Christian communities that were far less affected by anti-Assyrian sentiments, despite the close historical connections between the groups. Apart from the fact that these Christians were not refugees, their choice not to identify as a separate national group certainly made a difference, of which the missionaries apparently became more and more aware. The missionaries furthermore realized over time that the Assyrians in Iraq would permanently stay in Iraq and that the Assyrians’ opposition against the government would be counterproductive. In this, they were supported by a change in modern humanitarian thought, which since the late 1920s stressed good relationships with local authorities by not resisting their policies and nationalist aspirations. It was also in line with new currents in American Protestantism that showed a growing appreciation of Islam, which might explain the greater degree of trust in the Iraqi government. The change in attitude of the missionaries of the United Mission was therefore consistent with the development of their policy in the region towards pragmatic accommodation. In addition, the fact that the missionaries were of the opinion that the Assyrians could have been in a better situation if their patriarch had supported integration in Iraq as loyal citizens instead of working against it has certainly influenced their attitude. The Assyrians they worked with may nonetheless have interpreted the sudden lack of support as an act of betrayal.

